# Seasonal blooms of *Synechococcus* in a temperate semi-enclosed bay: linking community succession to thermal and nutrient regimes

**DOI:** 10.3389/fmicb.2025.1650890

**Published:** 2025-08-05

**Authors:** Xiaokun Ding, Guannan Wu, Di Zhang, Long Yang, Aobo Wang, Sheng Li, Li Gao, Zhengguo Cui, Tao Jiang

**Affiliations:** ^1^School of Ocean, Yantai University, Yantai, China; ^2^School of Hydraulic Engineering, Ludong University, Yantai, China; ^3^Key Laboratory of Sustainable Development of Marine Fisheries, Ministry of Agriculture, Yellow Sea Fisheries Research Institute, Chinese Academy of Fishery Sciences, Qingdao, China

**Keywords:** *Synechococcus* bloom, flow cytometry, *rpoC1* gene, community, temperature threshold, nutrient inputs

## Abstract

*Synechococcus* is a key picocyanobacterium in coastal ecosystems, yet its seasonal bloom dynamics and environmental responses remain unclear in temperate coastal seas. Here, we integrated flow cytometry and *rpoC1* gene analysis to investigate its bloom development and community succession in Laizhou Bay, based on 3 years of 10 seasonal surveys and a year-long monthly observation at a fixed station. *Synechococcus* blooms reached their peak in summer (up to 10^6^ cells mL^−1^), particularly in the southern part of the bay, with high abundances in autumn as well. Phycoerythrin-rich *Synechococcus* consistently dominated the community (>70%), especially during autumn blooms. Genetic analyses revealed that summer-autumn blooms harbored high clade diversity (S5.1 II, III, V, and VII), whereas winter and spring communities were simpler, dominated by S5.1 I and IV. Notably, S5.2. VIII gradually increased in relative abundance during bloom development, exceeding 50% in late autumn. Temperature emerged as the primary regulator of *Synechococcus* dynamics, with cell abundance increasing exponentially with rising temperature. Bloom events were consistently triggered above 26°C. In addition, external nutrient inputs, particularly riverine pulses accumulating from summer to autumn, contributed to *Synechococcus* bloom persistence and genetic diversification. This study provides valuable insights into the mechanisms regulating *Synechococcus* blooms and offers a methodological framework for understanding and predicting microbial community responses to the combined effects of climate change and anthropogenic disturbances in coastal ecosystems.

## 1 Introduction

*Synechococcus*, a ubiquitous and ecologically significant genus of marine picocyanobacteria, plays a pivotal role in oceanic primary production and biogeochemical cycling, particularly in coastal seas (Doré et al., 2022; Kling et al., 2023). This genus exhibits remarkable physiological and genetic diversity, allowing it to occupy a wide range of ecological niches along gradients of temperature, salinity, light, and nutrient availability (Flombaum et al., 2013; Li et al., 2024). *Synechococcus* populations are typically categorized into pigment types based on their phycobiliprotein composition, namely phycocyanin (PC) and phycoerythrin (PE), and into phylogenetic clades using marker genes such as 16S rRNA, *petB*, and *rpoC1* (Ahlgren and Rocap, 2006; Mazard et al., 2012). Among these, the *rpoC1* gene has proven to be a particularly robust phylogenetic marker, offering higher resolution in distinguishing *Synechococcus* lineages compared to traditional markers (Mühling et al., 2006). These molecular and physiological classifications are closely linked to ecological strategies and environmental preferences, making *Synechococcus* an ideal model organism for studying microbial community dynamics and environmental adaptation.

Globally, *Synechococcus* has gained prominence as a key organism in harmful algal blooms, particularly in coastal regions experiencing eutrophication and climate change (Paerl et al., 2020; Glibert et al., 2023). These blooms are increasingly recognized for their potential ecological disruptions, such as oxygen depletion, harmful secondary metabolites, and alterations to food webs (Phlips et al., 1999; Sunda et al., 2006). In temperate coastal seas, *Synechococcus* blooms are often triggered by seasonal nutrient inputs and environmental changes (Xia et al., 2015; Jiang et al., 2016; Li et al., 2019; Wang et al., 2022a), yet the mechanisms underlying their bloom dynamics and community succession, particularly in relation to nutrient availability and temperature, remain uncertain and require further investigation.

Recent studies have increasingly focused the diversity and seasonal dynamics of *Synechococcus* in various marine systems, including temperate estuaries and open oceans (e.g., Larkin et al., 2020; Wang et al., 2022a; Yeh and Fuhrman, 2022; Harding et al., 2025). However, less attention has been paid to *Synechococcus* ecology in marginal seas that are subject to large and highly variable riverine discharge. These systems, such as Laizhou Bay, are characterized by pronounced seasonal fluctuations in salinity, nutrient concentrations, and water column stratification, driven by freshwater inflows and regional hydrodynamics (Ding et al., 2020; Yu et al., 2020). Such complex and dynamic environments may support distinct patterns of *Synechococcus* bloom development and community succession that remain poorly characterized.

**Figure 1:**
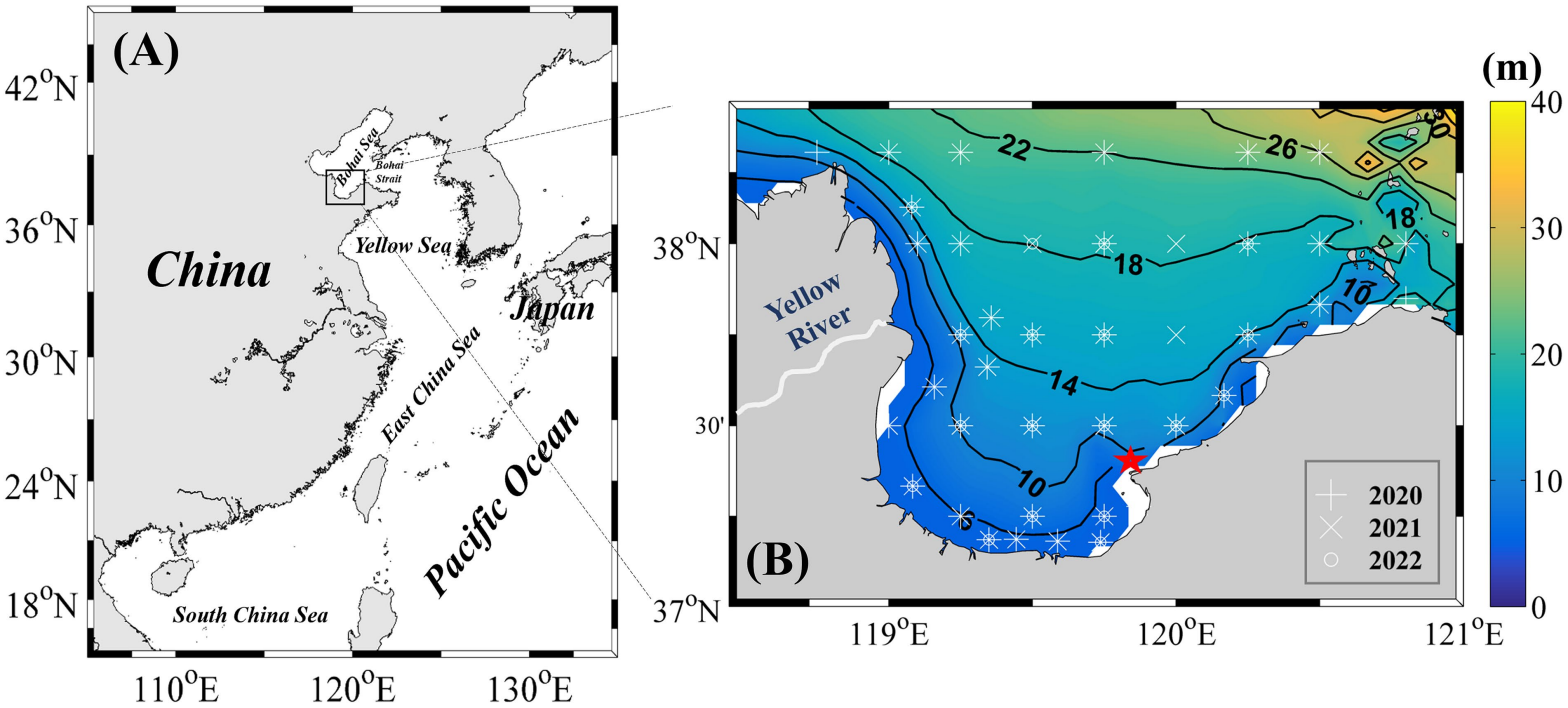
**(A)** Geographic location of Laizhou Bay. **(B)** Distribution of sampling stations from 2020 to 2022. The red pentagram indicates key sites that were sampled monthly from July 2021 to June 2022.

Laizhou Bay, a semi-enclosed embayment in the Bohai Sea of northern China (Figure 1), provides a representative setting for studying *Synechococcus* succession under strong environmental gradients and anthropogenic pressures. The bay is affected by discharges from the Yellow River and modulated by regional hydrodynamics, leading to pronounced seasonal variations in nutrient availability and the formation of a low-salinity surface layer that suppresses vertical mixing and induces transient stratification during periods of high discharge (Liu, 2015; Luo et al., 2021; Ding et al., 2024). Notably, *Synechococcus* regularly dominates the picophytoplankton community in this region, with bloom events frequently observed during warm months (Wang et al., 2022b; Wang et al., 2023), yet data on bloom dynamics, genetic composition, and ecological controls remain scarce.

In this study, we present a high-resolution analysis of *Synechococcus* biomass, pigment-type composition (PC and PE), and genotypic structure in Laizhou Bay, integrating flow cytometry and *rpoC1* gene sequencing. Our dataset encompasses ten bay-wide seasonal surveys conducted from 2020 to 2022, supplemented by monthly monitoring at a fixed station from July 2021 to June 2022. This multi-year comprehensive observations allows us to: (i) characterize the seasonal patterns of *Synechococcus* abundance and bloom development; (ii) link pigment types and genotypes to key environmental variables; and (iii) explore the mechanisms underlying *Synechococcus* bloom initiation, persistence, and ecological differentiation. Our findings provide new insights into the bloom ecology of *Synechococcus* in temperate coastal seas and contribute to the refinement of coastal biogeochemical and microbial models under climate change scenarios.

## 2 Materials and methods

### 2.1 Sample collection

From 2020 to 2022, ten seasonal research cruises were conducted in Laizhou Bay. Sampling was performed in May, August, and October 2020; January, March, August, October, and December 2021; and August and October 2022. Each year, more than 40 sampling stations were surveyed, with their locations shown in Figure 1B. In addition, a fixed coastal station in the eastern part of Laizhou Bay (Figure 1B) was monitored monthly from July 2021 to June 2022, yielding 12 high-frequency observations. Although this nearshore station does not fully capture the spatial heterogeneity of the entire bay, its time-series data provide valuable temporal resolution that complements the broader seasonal spatial surveys, thereby facilitating a more detailed understanding of the seasonal dynamics and succession of *Synechococcus* populations.

Surface seawater (0.5 m depth) temperature, salinity and dissolved oxygen (DO) were measured *in situ* using a YSI 556 multiparameter probe (Yellow Springs Instruments, United States), and pH was determined onboard using a glass electrode. For nutrient analysis, 300 mL of seawater was filtered through GF/F filters (0.7 μm pore size, Whatman) and stored at −20°C until analysis. Concentrations of nitrate, nitrite, ammonium, dissolved inorganic phosphorus (DIP), and dissolved silica (DSi) were determined using a continuous flow analyzer (Seal QuAAtro, Germany) following Jiang et al. (2022). Dissolved inorganic nitrogen (DIN) was calculated as the sum of nitrate, nitrite, and ammonium. For chemical oxygen demand (COD) analysis, 250 mL of seawater was collected and immediately frozen at −20°C. COD was determined using the standard dichromate oxidation method with reflux digestion and titration with potassium dichromate (Wei et al., 2022). For chlorophyll *a* (Chl-a) analysis, 1 L of seawater was filtered through GF/F filters and stored in liquid nitrogen. Chl-a concentrations were subsequently quantified using high-performance liquid chromatography.

### 2.2 Flow cytometry

Seawater samples were pre-filtered through a 20 μm mesh and fixed with 2% paraformaldehyde in 2 mL cryovials, kept in the dark for 15 min, and then rapidly frozen in liquid nitrogen. PE-rich and PC-rich type *Synechococcus* populations were identified using a Becton Dickinson FACSCalibur flow cytometer equipped with dual lasers (488 nm and 635 nm), following the procedure of Liu et al. (2014). Four fluorescence channels were analyzed: FL1 (530/30 BP), FL2 (585/42 BP), FL3 (670 LP), and FL4 (661/16 BP). PE-rich cells were identified by strong orange fluorescence (FL2), while PC-rich type cells were distinguished by their red fluorescence (FL4).

### 2.3 DNA extraction, amplification, and *rpoC1* sequencing

Environmental DNA was extracted using an enzyme/phenol-chloroform protocol (Riemann et al., 2008), and eluted in TE buffer (10 mM Tris, 1 mM EDTA, pH 8.0). The *rpoC1* gene of *Synechococcus* was amplified via nested Polymerase Chain Reaction (PCR) following the protocol of Mühling et al. (2006). The first round employed primers *rpoC1*-N5 and *rpoC1*-C, and the second round used barcoded primers *rpoC1*-39F and *rpoC1*-462R, which target a ~ 400 bp fragment of the *rpoC1* gene.

To ensure spatial representativeness while maintaining feasibility, 9 to 13 samples were selected from each cruise for *rpoC1* analysis. Selection was based on spatial distribution and prevailing weather conditions during each cruise. Successful *rpoC1* amplification was achieved for nine of the ten seasonal cruises; no usable sequences were obtained from the December 2021 cruise due to sample degradation.

PCR products were gel-purified (Qiagen, Germany), quantified using the Quant-iT picoGreen double stranded DNA (dsDNA) assay kit (Invitrogen, USA), pooled in equimolar concentrations, and sequenced on a Roche GS Junior 454 pyrosequencing platform (Xia et al., 2015). Raw reads were quality-filtered using mothur (v1.35.1; Schloss et al., 2009), retaining sequences between 300 and 500 bp in length with average quality scores > 20. Denoising was performed with shhh.seqs using a sigma value of 0.01, and chimeras were removed via chimera.uchime (Schloss et al., 2011). High-quality sequences were then aligned and compared to a curated reference database of *Synechococcus rpoC1* sequences. Each sequence was assigned to a known phylogenetic clade based on BLAST results (identity > 90%, E-value < 0.01). Sequences with < 90% identity were designated as unclassified (Xia et al., 2015). This genotype-level classification enabled detailed analysis of *Synechococcus* community structure and seasonal succession.

In this study, an exact amplicon sequence variant (ASV) approach was applied to the *rpoC1* sequence data using the DADA2 pipeline, enabling single-nucleotide resolution without reliance on arbitrary clustering thresholds (Callahan et al., 2017). For phylogenetic analysis, representative ASV sequences were aligned using a 90% similarity cutoff, a common practice for functional genes with high sequence divergence such as *rpoC1* (Turk-Kubo et al., 2015). This approach preserves the high-resolution taxonomic information inherent to ASVs while allowing robust tree construction. All downstream diversity and community composition analyses were conducted at the ASV level to ensure analytical precision and comparability with current microbial ecological standards.

## 3 Results

### 3.1 Spatiotemporal variation of environmental factors

**Figure 2:**
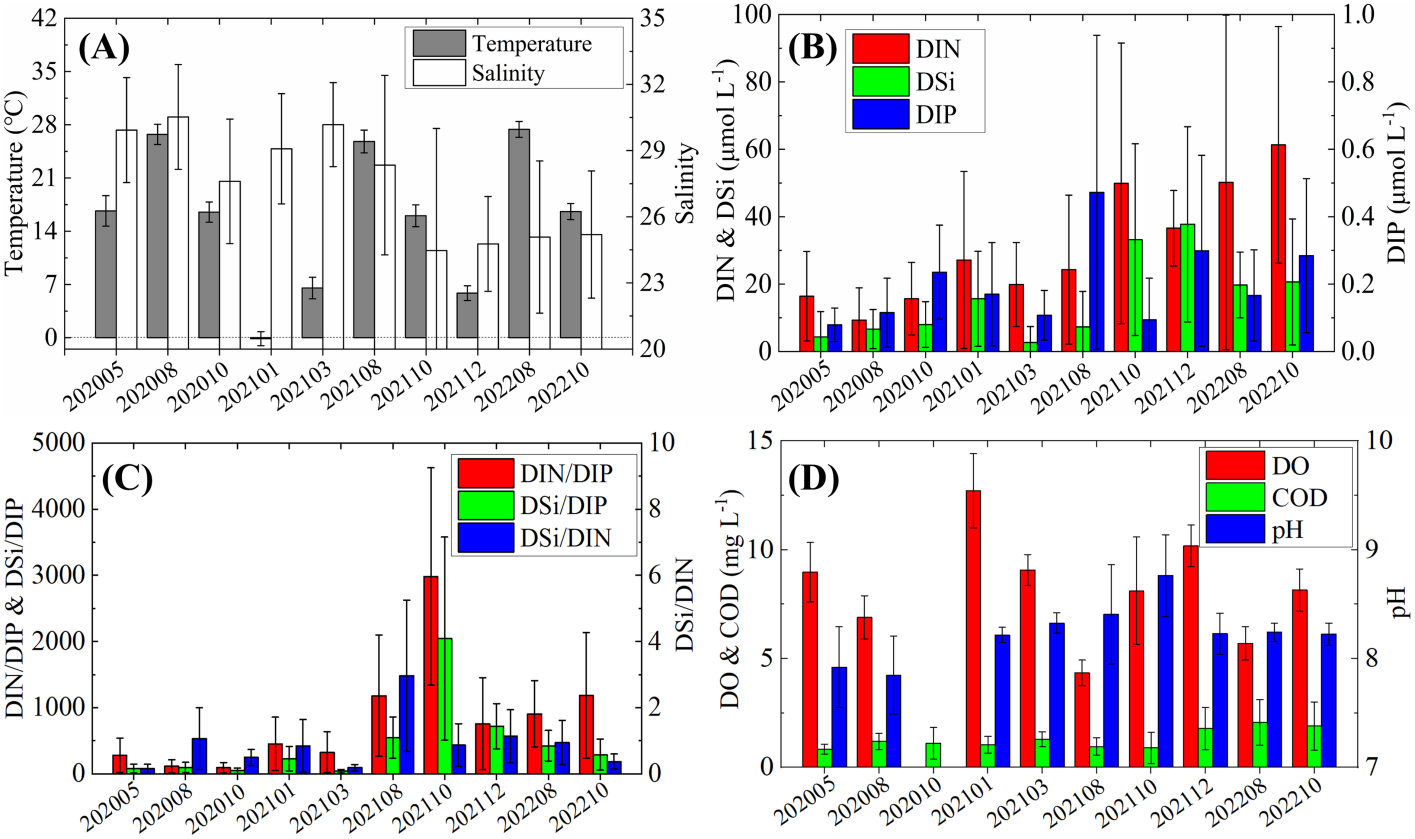
Temporal variations in environmental factors from 2020 to 2022. **(A)** Temperature and salinity; **(B)** nutrients concentrations; **(C)** nutrients ratios; **(D)** Dissolved oxygen (DO), chemical oxygen demand (COD), and pH.

From May 2020 to October 2022, ten surveys revealed pronounced temporal variability in the environmental conditions of Laizhou Bay (Figure 2). Surface water temperature exhibited a clear seasonal cycle, peaking in August (~ 28°C) and declining to around 17°C in May and October. Winter temperatures dropped to near 0°C (Figure 2A). Salinity was notably lower between October 2021 and October 2022, averaging around 25, with similarly low values (~ 27) also observed in October 2020.

DIN and DSi concentrations increased markedly in October 2021, more than doubling compared to earlier cruises (Figure 2B). In contrast, DIP remained consistently low (< 0.5 μmol L^−1^) throughout the study period, showing irregular fluctuations without a clear seasonal trend. The molar ratios of DIN/DIP and DSi/DIP were substantially elevated, far exceeding the Redfield ratio (N/*p* = 16), with values reaching ~ 3,000 and ~ 2000, respectively, in October 2021 (Figure 2C). The DSi/DIN ratio peaked in August 2021 (~ 3), suggesting a relative accumulation of silicate. DO concentrations were generally lower in summer, particularly in August, but never dropped to hypoxic levels (Figure 2D). COD showed a slight increase from December 2021 onwards, while pH exhibited notable fluctuations, ranging from a low of 7.8 in August 2020 to a high of 8.7 in October 2021.

### 3.2 Abundance and pigment types of *Synechococcus*

**Figure 3:**
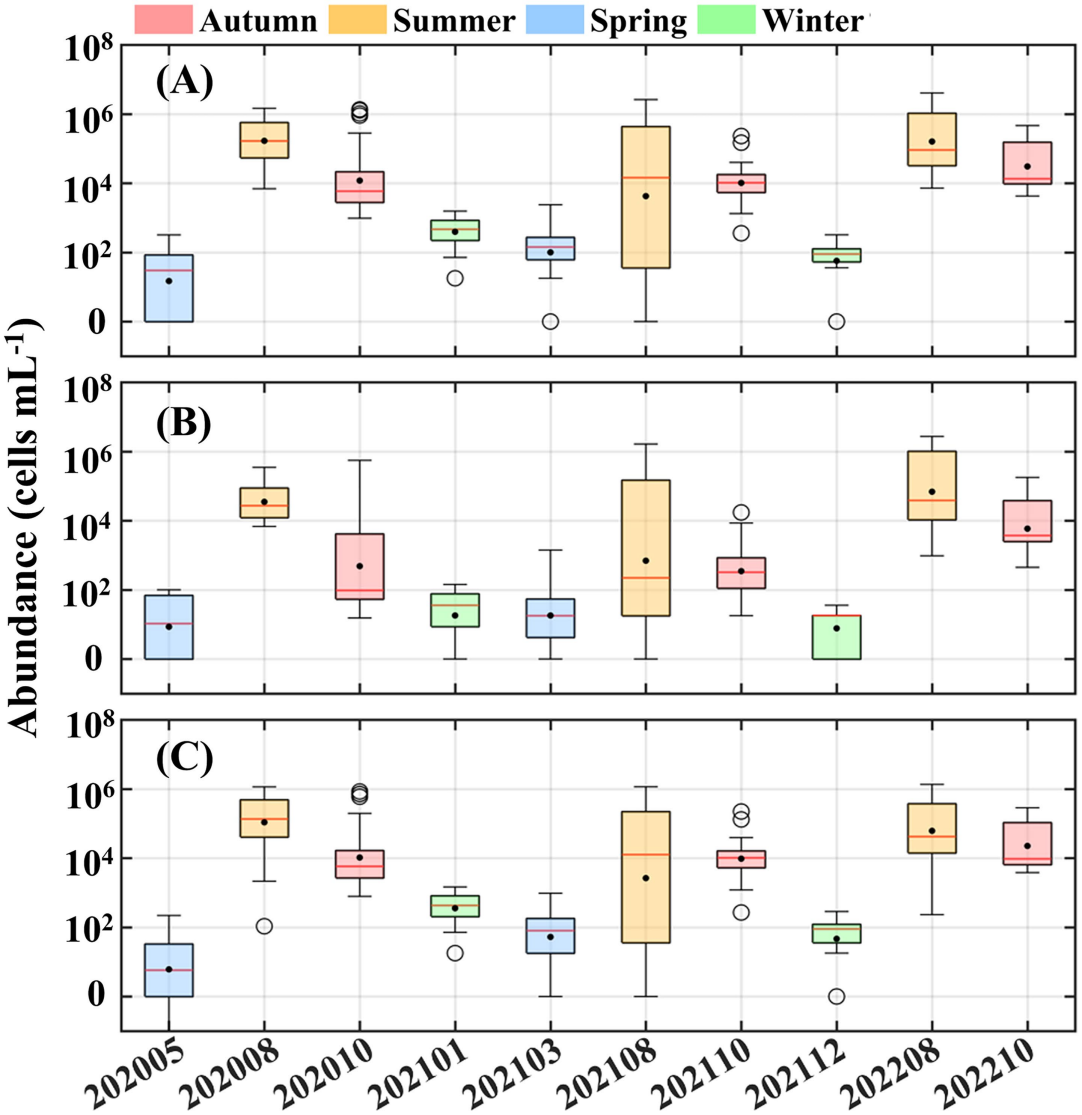
Variations in the cell abundance of **(A)** *Synechococcus*, **(B)** phycocyanin (PC)-rich type, and **(C)** phycoerythrin (PE)-rich type cells. Colored boxes represent four seasons classified by the corresponding sampling time. In each boxplot, solid dot represents mean value, three horizontal lines of box represent first quartile, median, and third quartile, respectively. Black circles refer to the abnormal values.

The cell abundance of *Synechococcus* exhibited pronounced seasonal variation, with significantly higher value in summer and autumn than in winter and spring (Figure 3). Peak abundances reached up to ~ 10^6^ cells mL^−1^ in August, whereas values typically remained below 100 cells mL^−1^ during colder seasons. Both PC-rich type and PE-rich type *Synechococcus* displayed similar seasonal trends; however, PE-rich type cells consistently dominated, with abundances approximately one order of magnitude higher than PC-rich type cells.

**Figure 4:**
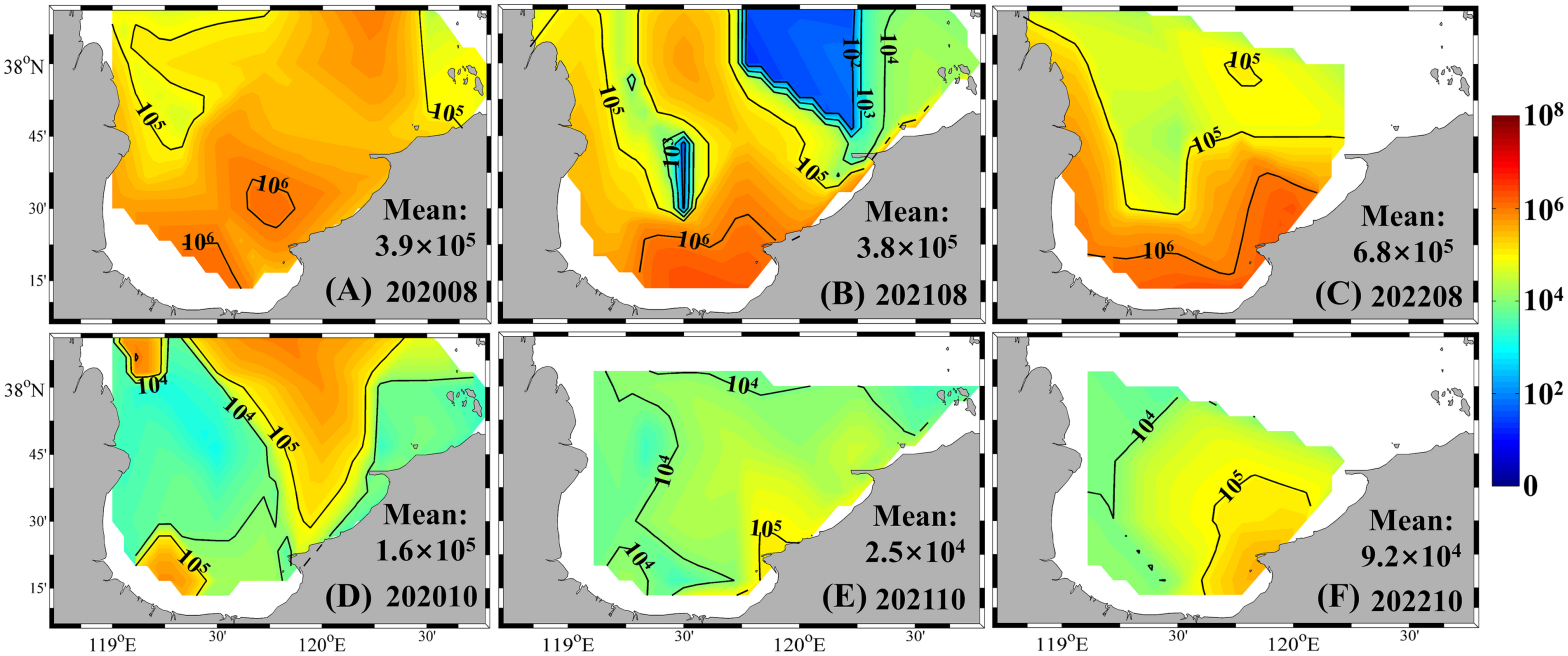
*Synechococcus* cell abundance in summer and autumn from 2020 to 2022 (unit: cells mL^−1^). **(A)** August 2020; **(B)** August 2021; **(C)** August 2022; **(D)** October 2020; **(E)** October 2021; **(F)** October 2022.

Horizontal distributions during summer and autumn further illustrated these seasonal dynamics (Figure 4). In summer, *Synechococcus* abundance decreased northward from the southern bay, where concentrations exceeded 10^6^ cells mL^−1^, to values below 10^5^ cells mL^−1^. Notably, a localized low-abundance patch (<10^3^ cells mL^−1^) was observed in central to northern Laizhou Bay in August 2021. In autumn, high-abundance zones (>10^5^ cells mL^−1^) emerged in both the northern and southern regions, while most other areas showed moderate levels (~10^4^ cells mL^−1^). In contrast, spatial variability was minimal in winter and spring, with nearly all stations recording abundances below 10^3^ cells mL^−1^ (Supplementary Figure S1).

**Figure 5:**
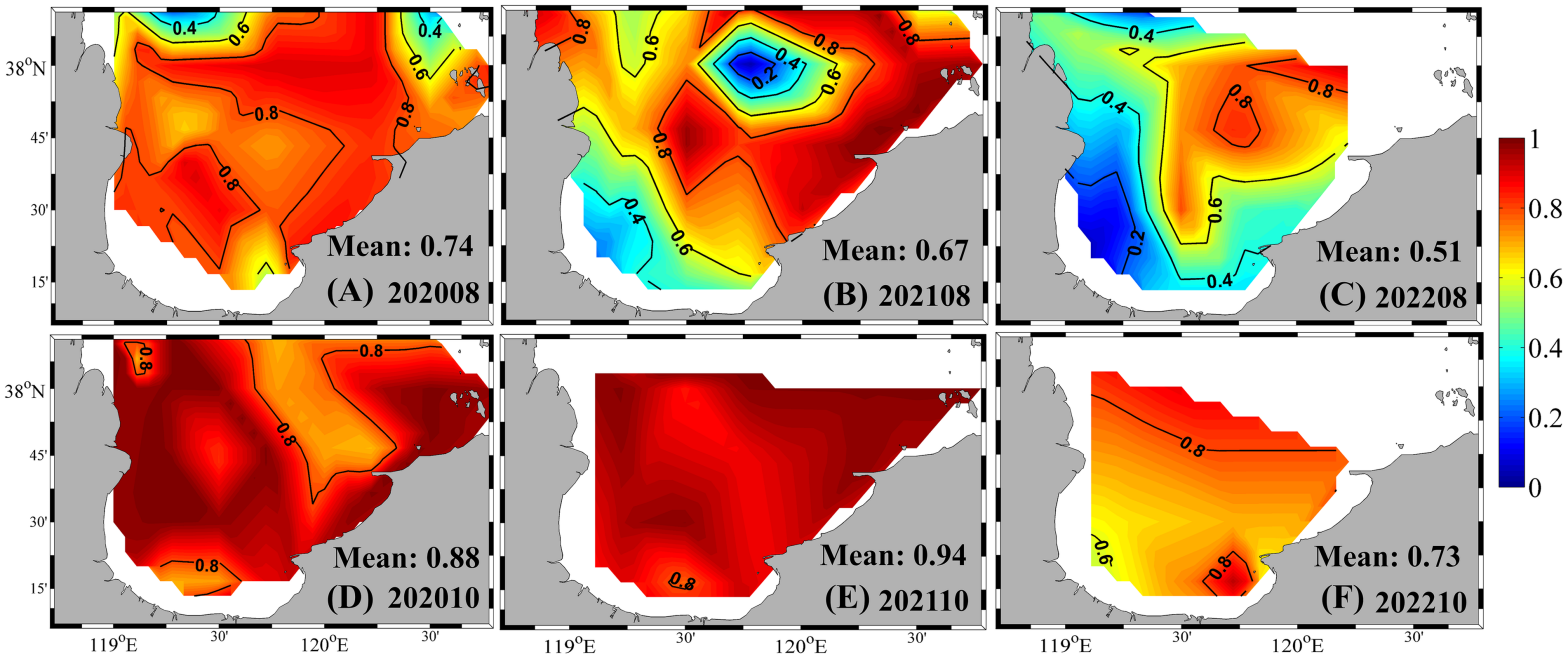
Proportion of PE-rich type abundance relative to total *Synechococcus* in summer and autumn from 2020 to 2022. **(A)** August 2020; **(B)** August 2021; **(C)** August 2022; **(D)** October 2020; **(E)** October 2021; **(F)** October 2022.

PE-rich type *Synechococcus* consistently dominated the community composition across seasons. In summer 2020, PE-rich type cells accounted for over 70% of total *Synechococcus* abundance throughout most of the bay (Figure 5). In summer 2021, their contribution remained high (> 60%) across much of the area, although it dropped below 40% in parts of the southwestern and central-northern bay. In summer 2022, PE-rich type *Synechococcus* comprised approximately half of the total abundance, increasing from ~ 20% in the southwest to ~ 80% in the northeast. During autumn, PE-rich type cells remained the dominant pigment type, contributing 88, 94, and 73% to the total abundance in 2020, 2021, and 2022, respectively. They also prevailed in winter and spring, except in May 2020, when their contribution dropped to 29% (Supplementary Figure S2).

### 3.3 Genotypic composition and spatiotemporal distribution of *Synechococcus*

A total of 14 *Synechococcus* clades were identified across the ten cruises and fixed-point time series, including subclusters within clade S5.1 (S5.1. I – S5.1. IX, S5.1. XV), as well as clades S5.2 and S5.3. Among them, clade S5.1. I is typically associated with cold or temperate nutrient-rich coastal waters, while S5.1. II and S5.1. III are thermophilic ecotypes that typically dominate in warm surface layers. Clade S5.1. V has been observed in warm-temperate and estuarine environments. Ecological characteristics of all identified clades are provided in Supplementary Table S1.

**Figure 6:**
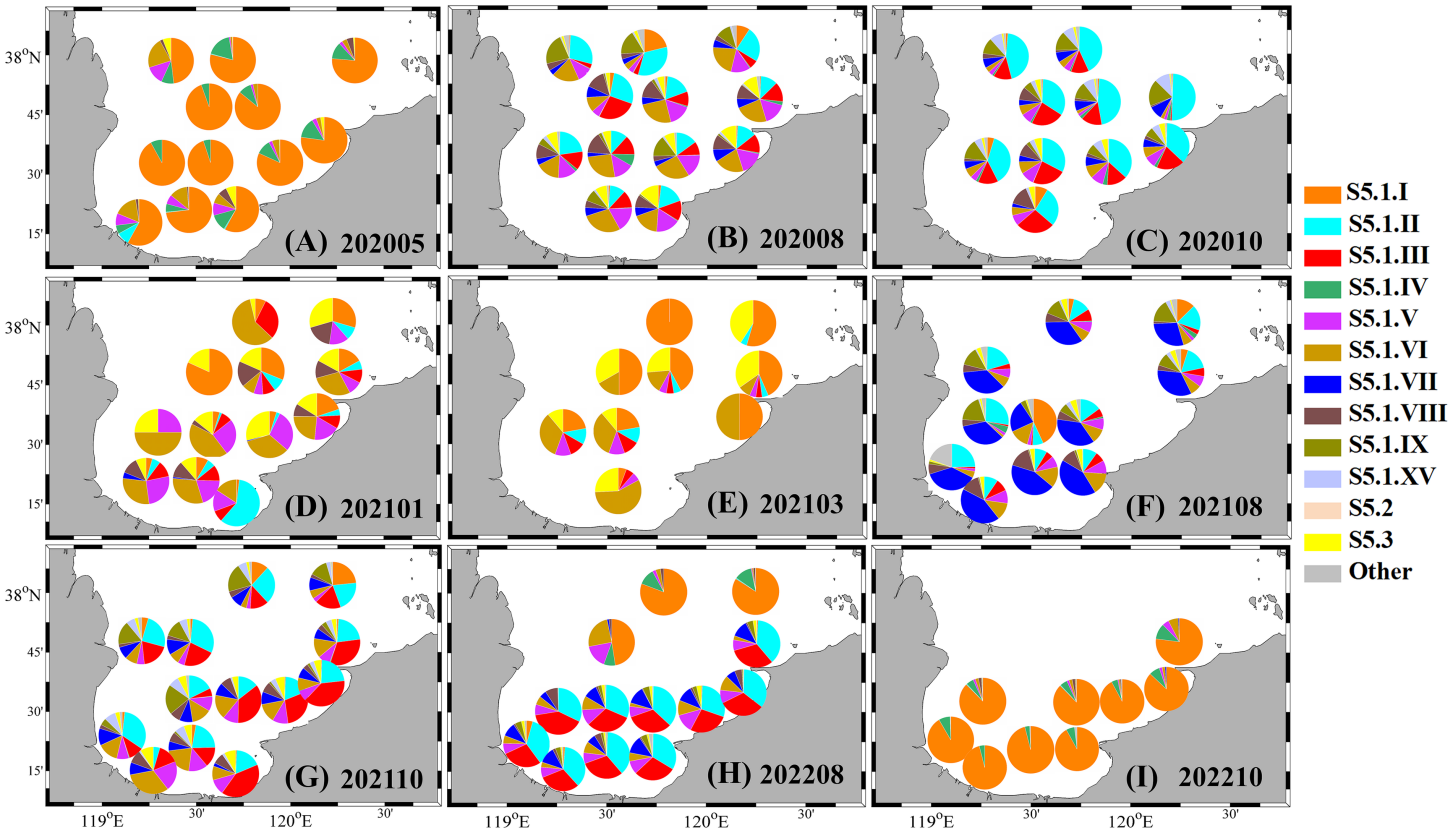
Spatiotemporal variations in the genotypic composition of *Synechococcus* from 2020 to 2022. **(A)** May 2020; **(B)** August 2020; **(C)** October 2020; **(D)** January 2021; **(E)** March 2021; **(F)** August 2021; **(G)** October 2021; **(H)** August 2022; **(I)** October 2022.

In spring 2020, clade S5.1. I overwhelmingly dominated the assemblage, accounting for over 50% of sequences at all stations, and exceeding 80% in central bay areas (Figure 6A). During periods of *Synechococcus* bloom, genotypic diversity increased substantially. As shown in Figures 6B,C, in August and October 2020, multiple clades such as S5.1. II, III, V, VI, VII, VIII, and IX co-occurred, with the first four clades contributing substantially to the assemblage (> 50%). In summer and autumn of 2021, clades S5.1. VII and S5.1. III increased notably, each contributing approximately 30% to the total composition, while overall genotypic diversity remained high (Figures 6F,G). In August 2022, clade S5.1. I once again became dominant (> 70%) in northern Laizhou Bay, whereas other regions were primarily occupied by S5.1. II and S5.1. III (Figure 6H). By October 2022, the assemblage across the entire bay was again overwhelmingly dominated by S5.1. I (> 80%), accompanied by the reappearance of clade S5.1. IV (Figure 6I). In colder months (January and March 2021), the community structure shifted, with clades S5.1. I, V, VI, and S5.3 representing the main genotypic components (Figures 6D,E).

### 3.4 Monthly dynamics of *Synechococcus* at the fixed station

**Figure 7:**
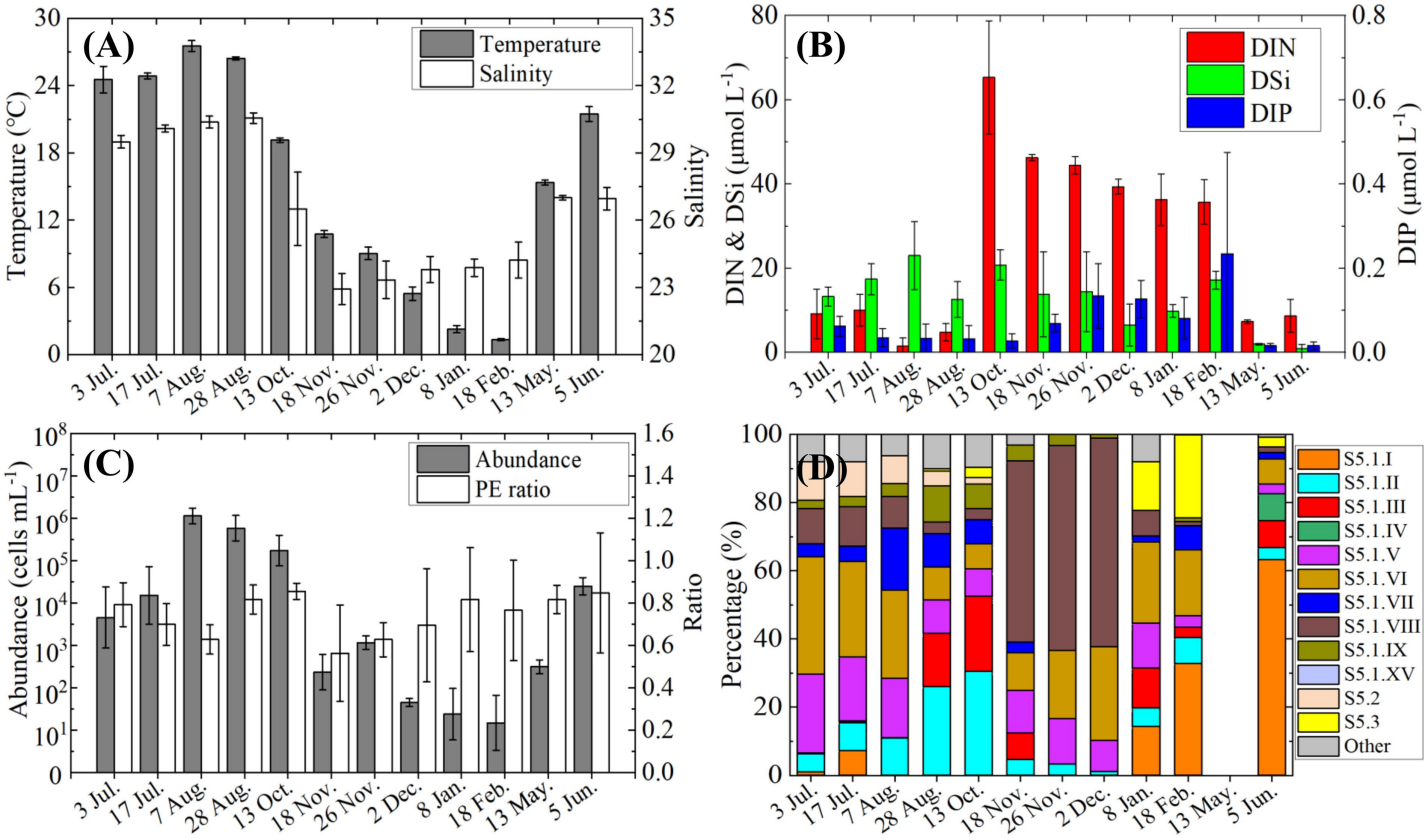
Variations in environmental conditions and *Synechococcus* characteristics at the fixed station from July 2021 to June 2022, **(A)** temperature and salinity; **(B)** nutrients concentrations; **(C)** *Synechococcus* cell abundance and the ratio of PE-rich type to total *Synechococcus*; **(D)** relative genotypic composition of *Synechococcus*.

Monthly observations at the fixed station from July 2021 to June 2022 revealed clear seasonal patterns in environmental conditions, *Synechococcus* abundance, pigment types, and genotypic composition (Figure 7).

Environmental variables showed strong seasonal variation (Figures 7A,B). Sea surface temperature was above 24°C in July–August, dropped steadily to ~2°C by February, and then increased to 15°C in May and 21°C in June. Salinity also peaked in July–August (> 29), decreased from October onward, and remained below 25 from November through February before recovering to ~27 in late spring. DIN concentrations rose sharply in October (>60 μmol L^−1^) and stayed elevated (>35 μmol L^−1^) until February. DIP remained low throughout the year (<0.2 μmol L^−1^), except for a moderate increase in February. DSi was relatively high (>20 μmol L^−1^) in August and October, fluctuated around 10 μmol L^−1^ for most of the year, and dropped below 5 μmol L^−1^ in May and June.

A pronounced bloom of *Synechococcus* began in July, with cell densities exceeding 10^5^ cells mL^−1^ from August to October (Figure 7C). Following the bloom, abundance declined sharply in November and remained below 10^3^ cells mL^−1^ through the following May, before increasing again in June (>10^4^ cells m^−1^). Throughout the year, PE-rich type *Synechococcus* consistently dominated the community, accounting for over 70% of total abundance, except in November when its relative contribution slightly declined.

Genotypic succession accompanied seasonal shifts in abundance (Figure 7D). In the early *Synechococcus* bloom stage (July), clades S5.1. V and VI were prominent, each comprising over 20% of the community. However, their proportions declined during peak bloom months (August and October), when S5.1. II and S5.1. III increased markedly, reaching 30 and 20%, respectively. During November and December, S5.1. VIII became dominant (>50%), along with a rise in S5.1. VI (~20%). From January to June, clade S5.1. I gradually increased and became the predominant genotype by June (>60%).

### 3.5 *Synechococcus*–environment relationships

**Figure 8:**
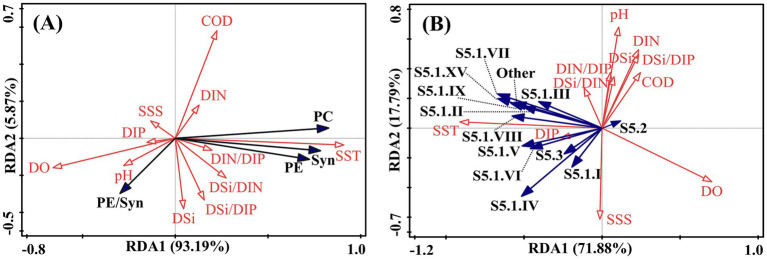
Redundancy analysis (RDA) biplot showing the relationship between **(A)** *Synechococcus* pigment types and **(B)** genotypic composition and environmental variables.

Redundancy analysis (RDA) based on data from ten cruises revealed the environmental drivers shaping *Synechococcus* abundance and community composition (Figure 8). Total *Synechococcus* abundance, as well as PC- and PE-rich type populations, showed strong positive correlations with SST, and negative correlations with DO and pH. However, the relative proportion of PE-rich type cells exhibited a negative association with both SST and COD, suggesting a potential shift in pigment-type dominance under warmer, more eutrophic conditions. Major genotypes such as S5.1. II, VII, VIII, and IX were positively correlated with SST and negatively correlated with DO, indicating temperature-driven niche preferences. In contrast, correlations between *Synechococcus* dynamics and nutrient concentrations or salinity were generally weak.

These findings were further supported by the monthly time series (Figure 7), where peak *Synechococcus* blooms consistently coincided with elevated temperature. Notably, although temperature was similar in October and May, *Synechococcus* abundance was markedly higher in October, which is further discussed in Section 4.2.

## 4 Discussion

### 4.1 Temperature as a key driver of *Synechococcus* dynamics

Our results demonstrate that temperature plays a pivotal role in shaping both the abundance and community composition of *Synechococcus* in Laizhou Bay. Throughout the seasonal surveys, *Synechococcus* consistently reached bloom-level densities from summer to mid-autumn (August and October), which coincided with the high seawater temperatures recorded (Figure 2A). Redundancy analysis further confirmed significant positive correlations between temperature and total *Synechococcus* abundance, as well as with several dominant pigment types and genotypic clades (Figure 8). These patterns are consistent with previous findings that temperature is a primary environmental factor governing the growth and distribution of picocyanobacteria in marine environments (Zwirglmaier et al., 2008; Mackey et al., 2013; Varkey et al., 2016).

**Figure 9:**
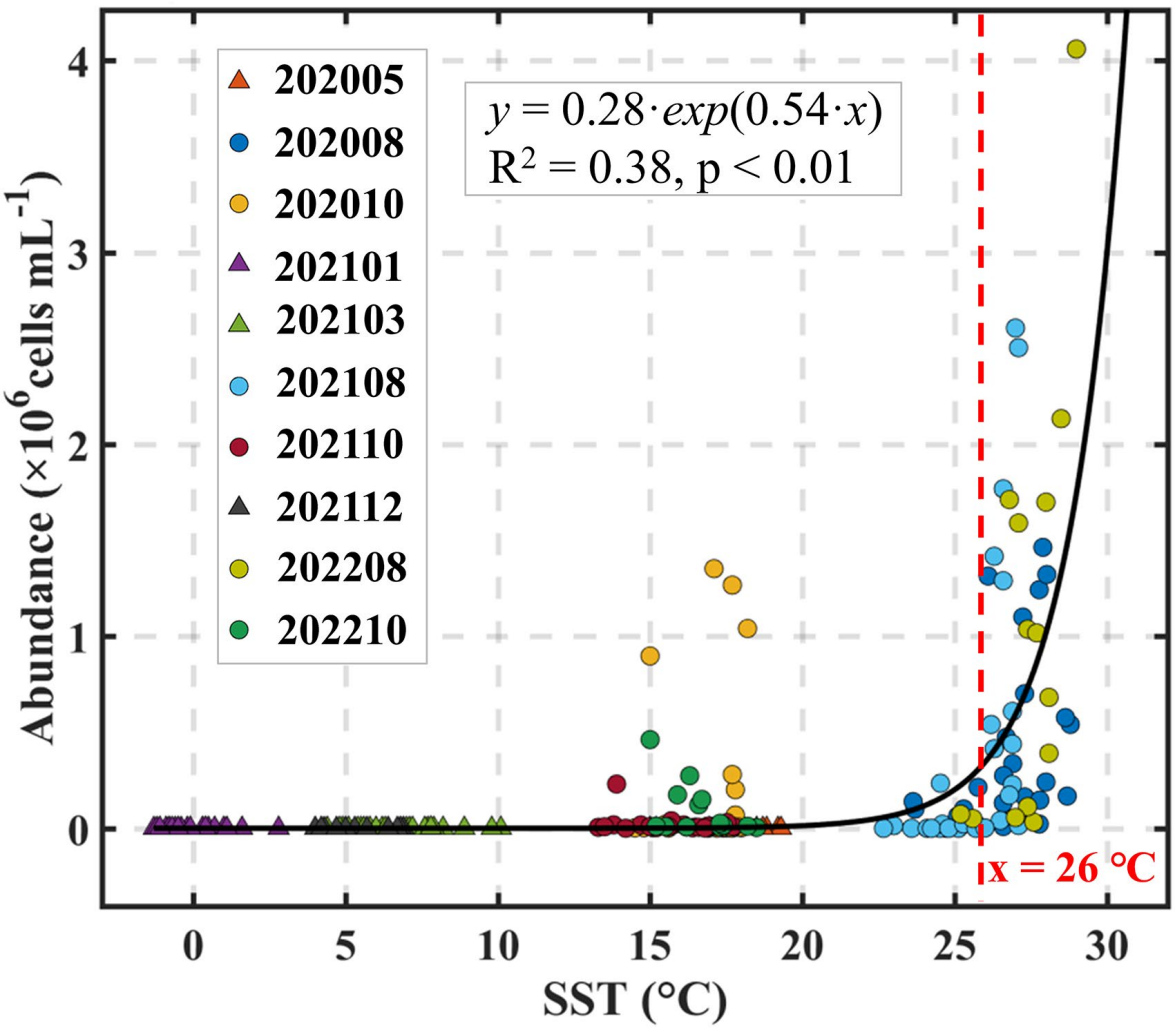
Relationship between *Synechococcus* abundance and sea surface temperature (SST) during the period of 2020–2022.

To further elucidate the mechanistic basis of this temperature control, we examined the direct relationship between seawater temperature and *Synechococcus* abundance. The analysis revealed a strong exponential increase in cell density with rising temperature (Figure 9), consistent with the well-established exponential dependence of biogeochemical and metabolic rates on temperature (Eppley, 1971; Sanz-Lázaro et al., 2011). Notably, our data revealed that *Synechococcus* abundance began to increase more rapidly around 26°C, suggesting this value may represent an approximate thermal inflection point under the specific environmental conditions of Laizhou Bay (Figure 9). This suggests that 26°C may represent a critical bloom-initiation temperature under the specific environmental conditions of Laizhou Bay. We emphasize that this threshold was derived from statistical fitting and should not be interpreted as a strict *Synechococcus* bloom-initiation criterion. Indeed, time-series observations at the fixed station (e.g., October 2021) recorded high *Synechococcus* abundance under slightly cooler temperatures (Figure 7), likely influenced by lag effects or concurrent nutrient availability.

Threshold-type responses of cyanobacteria to environmental factors have been observed in various contexts, including light intensity, depth, and Chl-a concentrations (Zinser et al., 2007; Cram et al., 2015; Yeh and Fuhrman, 2022). Specifically, temperature thresholds triggering *Synechococcus* blooms have been reported in other marine systems, although the precise inflection points can vary depending on local ecological settings ranging from below 15°C to ~ 25°C in different studies (e.g., Johnson et al., 2006; Larkin et al., 2020). While the identification and mechanistic understanding of such threshold behaviors remain areas for further systematic investigation, our findings provide ecologically relevant evidence of a thermal inflection point under eutrophic semi-enclosed bay conditions. This contributes to the broader understanding of *Synechococcus* bloom dynamics and may inform predictive models in temperate coastal ecosystems facing future climate warming.

Temperature appeared to structure community composition by selecting for genotypes with distinct thermal niches. For example, several PE-rich type lineages (e.g., clades S5.1. II, III, and VII) were positively associated with temperature (Figure 8B), implying thermal differentiation among subpopulations. These observations are in line with the concept of “thermal ecotypes” previously reported in picocyanobacterial assemblages (Johnson et al., 2006; Kling et al., 2023), whereby even closely related genotypes exhibit divergent thermal performance curves. Previous studies have shown that clade S5.1. I typically inhabits cold or temperate waters and can occur in both coastal and oceanic environments (Farrant et al., 2016; Sohm et al., 2016), which supports our observation of its dominance during the spring (May 2020) and autumn (October 2022) cruises. Furthermore, elevated summer temperatures may enhance water column stratification, leading to reduced mixing and prolonged light exposure in surface layers, which benefits phototrophic organisms like *Synechococcus*, particularly in turbid coastal settings (Behrenfeld et al., 2006; Fischer et al., 2014).

Although the total abundance of *Synechococcus* increased with temperature, the relative proportion of PE-rich type cells exhibited a negative correlation with sea surface temperature (Figure 8A). This apparent decoupling between absolute growth and pigment-type composition suggests more complex regulatory dynamics. One possible explanation is that different pigment types possess distinct thermal optima or light-harvesting efficiencies, leading to shifts in competitive interactions under warming conditions (Six et al., 2007; Pittera et al., 2018). Alternatively, temperature may interact with other factors such as nutrient stress, pH, or grazing pressure, indirectly modulating pigment-type structure (Zwirglmaier et al., 2007; Grébert et al., 2018). These findings underscore the importance of jointly considering both abundance and community composition when evaluating picocyanobacterial responses to environmental drivers.

### 4.2 Nutrient availability and its seasonal modulation of *Synechococcus* blooms

*Synechococcus* exhibited apparent spatial distributions in both cell abundance and community composition during the bloom periods (Figures 4–6), even though seawater temperatures showed no obvious difference across these times. Notably, *Synechococcus* biomass in October was markedly higher than in May, despite comparable thermal conditions (Figure 7). These divergences highlight that temperature alone cannot fully account for bloom dynamics and underscore the importance of other regulatory factors, such as nutrient availability and ecological interactions, in shaping *Synechococcus* population responses.

#### 4.2.1 The role of luxury phosphorus uptake

Laizhou Bay is a eutrophic yet phosphorus-limited coastal system (Jiang et al., 2022), as evidenced by consistently elevated DIN/DIP and DSi/DIP ratios (>100) (Figure 2C). This severe nutrient imbalance implies intense biological phosphorus uptake and suggests that phytoplankton communities, including *Synechococcus*, are experiencing chronic phosphorus-limitation, particularly during periods of elevated DIN and DSi availability (Dyhrman et al., 2006; Jin et al., 2024). A key physiological adaptation that may explain the sustained proliferation of *Synechococcus* under such conditions is “luxury phosphorus uptake.” This process enables phytoplankton to assimilate DIP at rates far exceeding their immediate metabolic demands during brief windows of elevated phosphorus availability, such as following riverine pulses, sediment resuspension, or storm-driven mixing, and store excess DIP intracellularly in the form of polyphosphate granules (Rhee, 1973; Cembella et al., 1984). Laboratory studies have shown that DIP uptake rates under luxury conditions can increase by over tenfold, supporting prolonged growth even when external DIP levels subsequently decline (Solovchenko A. et al., 2019; Solovchenko A. E. et al., 2019). In Laizhou Bay, once internal phosphorus reserves are established, *Synechococcus* may maintain rapid cell division during DIP-depleted phases, effectively decoupling population growth from ambient phosphorus concentrations. This strategy provides a plausible explanation for the October biomass peak observed in our study (Figures 3, 4), as the community may have capitalized on earlier summer nutrient pulses to sustain autumn blooms (Mackey et al., 2009; Ding et al., 2024).

#### 4.2.2 Episodic nutrient pulses and external forcing in *Synechococcus* bloom regulation

The dynamic nature of nutrient cycling in coastal waters poses significant challenges for interpreting the role of nutrients using static concentration data. Nutrients, being non-conservative substances, are subject to rapid biological uptake, remineralization, and various source-sink processes (Liu et al., 2009; Ding et al., 2021). As such, measured concentrations often reflect the net result of complex biogeochemical transformations rather than the actual nutrient supply. In regions like Laizhou Bay, where riverine and submarine groundwater discharges deliver high loads of nutrients, phytoplankton abundance often shows stronger correlations with nutrient input fluxes than with ambient concentrations (Jiang et al., 2022; Ding et al., 2024). Our results demonstrate that *Synechococcus* consistently exhibited high biomass from August to October, even under low DIP concentrations (<0.05 μmol L^−1^) (Figures 4, 7), suggesting that episodic nutrient enrichment events may trigger and sustain blooms. Notably, the Water-Sediment Regulation of the Yellow River delivers large nutrient pulses to the Bohai Sea in early summer (Liu, 2015; Li et al., 2017). Additionally, extreme rainfall events, such as the autumn Yellow River flood in 2021, are known to generate substantial freshwater and nutrient influxes into Laizhou Bay (Ding et al., 2024).

Laizhou Bay is also characterized by weak hydrodynamic flushing, which prolongs the residence time of exogenous nutrients (Li et al., 2015; Ding et al., 2024). This residence effect contributes to the persistently elevated *Synechococcus* biomass observed between August and October each year (Figure 4). Studies have shown that nutrient pulses from riverine inputs can have prolonged effects on coastal phytoplankton communities, influencing bloom development well beyond the initial input event (Ge et al., 2020; Ding et al., 2024). Moreover, multiple terrestrial and atmospheric nutrient pathways (e.g., river runoff, submarine groundwater discharge, atmospheric deposition, and point-source effluents) co-occur in this semi-enclosed coastal system, each exhibiting distinct spatial and temporal dynamics (Ding et al., 2021). In addition, interannual differences in East Asian summer monsoon patterns can substantially modify surface circulation and nutrient transport pathways in Laizhou Bay (Liu et al., 2017; Ding et al., 2020). Consequently, *Synechococcus* bloom patterns in Laizhou Bay arise from a complex interplay between exogenous nutrient inputs, water retention, and physical forcing, underscoring the need for integrated monitoring of nutrient fluxes and physical processes to unravel bloom dynamics in such eutrophic coastal ecosystems.

### 4.3 Diversity and spatiotemporal dynamics of *Synechococcus*

Our results reveal pronounced spatiotemporal variability in *Synechococcus* community composition in Laizhou Bay, encompassing both pigment types and genotypes. PE-rich types consistently dominated throughout most of the year, accounting for over 70% of total abundance during bloom periods (Figure 5), with their dominance only weakening in early spring (e.g., ~29% in May 2020). This pattern aligns with observations from other coastal systems, where PE-rich type *Synechococcus* are favored under high-light, stratified conditions due to their efficient light-harvesting antennae and broad ecological plasticity (Liu et al., 2014; Wang et al., 2022b; Li et al., 2024). In contrast, PC-rich type *Synechococcus* are generally confined to estuarine and low-salinity environments rich in red light, and are rarely found in offshore waters (Wood et al., 1998). However, in Laizhou Bay, frequent Yellow River-derived freshwater and sediment inputs (Liu, 2015) likely create turbid, low-salinity coastal conditions that support PC-rich type populations. In fact, PC-rich type *Synechococcus* occasionally exceeded PE-rich types in southern coastal areas in summer (Figure 5), highlighting the complex niche partitioning shaped by freshwater influence and local optical properties.

Genotypic data further emphasize the seasonal succession within the *Synechococcus* assemblage. The S5.1 clade, widely distributed in coastal waters and comprising over 20 known sub-lineages (Li et al., 2024), was the most prevalent in our study. Clade S5.1. I, typically associated with cold or temperate nutrient-rich waters (Zwirglmaier et al., 2008; Sohm et al., 2016), dominated during early spring and winter (Figures 6, 7D). Interestingly, it also re-emerged as the dominant lineage in May 2020 and October 2022 despite relatively warm temperatures, suggesting that temperature alone does not fully control seasonal succession. Other ecological drivers, such as viral pressure, selective grazing, or legacy effects from earlier nutrient pulses, may also influence genotype dynamics. Similar patterns of S5.1. I dominance under warm conditions have been reported in other coastal systems, such as the western North Atlantic (Hunter-Cevera et al., 2016), Southern California Bight (Tai and Palenik, 2009) and the Bohai Sea (Li et al., 2024).

During summer and autumn blooms, community diversity increased markedly and was often dominated by clades S5.1. II, III, and VII (Figure 6). These lineages are known to prefer warm, nutrient-rich surface waters and may have competitive advantages such as rapid growth rates and efficient nutrient uptake mechanisms under stratified conditions (Post et al., 2011; Sohm et al., 2016). Similar seasonal shifts in genotypic composition have been reported in other temperate and subtropical coastal systems. For example, Larkin et al. (2020) and Yeh and Fuhrman (2022) documented positive correlations between clade II and temperature in the western North Atlantic and the San Pedro Channel, respectively. In contrast, Tai and Palenik (2009) observed a summer dominance of clades I and IV, with clades II and III remaining at low abundance, in the Southern California Bight. At our fixed station, a distinct genotypic succession was observed from clades II, III, and VI in July–August to clade VIII in August–October of 2021. These patterns likely reflect intra-clade competition, niche differentiation, or varying responses to shifting light and nutrient regimes during *Synechococcus* bloom development.

Compared to other eutrophic coastal systems such as Jiaozhou Bay or the Pearl River Estuary, where *Synechococcus* genotypic shifts are often driven by riverine inputs and salinity gradients (Xia et al., 2015; Li et al., 2024), Laizhou Bay appears to be more strongly influenced by internal processes such as benthic nutrient regeneration, thermal stratification, and biotic interactions. Specifically, factors like viral lysis, allelopathic inhibition, or grazing could differentially affect the fitness of specific genotypes, driving community restructuring during different seasons (Suikkanen et al., 2004; Wang and Chen, 2004; Labrie et al., 2013). Future studies incorporating metatranscriptomic profiling or physiological trait analyses under controlled conditions will be crucial for deciphering the mechanisms underlying clade-specific responses and ecological succession patterns.

### 4.4 Future perspectives: climate variability and modeling implications

The spatiotemporal dynamics of *Synechococcus* in coastal systems like Laizhou Bay are likely to become increasingly complex under the dual pressures of long-term climate warming and intensifying short-term weather extremes. Rising sea surface temperatures may progressively shift the competitive balance among picocyanobacterial clades, favoring thermophilic lineages (e.g., clade S5.1. II and III) with higher thermal optima and metabolic plasticity (Flombaum et al., 2013; Kling et al., 2023). Moreover, the increasing frequency of extreme precipitation events, driven by anthropogenic climate change (Shiu et al., 2012; Pendergrass, 2018), may trigger abrupt and episodic pulses of freshwater and nutrient influxes, thereby reshaping local salinity, stratification, and nutrient availability. Such stochastic perturbations could disrupt established seasonal succession patterns, favoring opportunistic or fast-growing *Synechococcus* ecotypes, or even facilitating transitions to alternate bloom regimes.

To anticipate and quantify these future shifts, an integrated approach combining *in situ* long-term monitoring, culture-based physiological assays, and trait-informed ecosystem modeling is crucial. Recent advances in isolating and culturing diverse *Synechococcus* clades under controlled conditions have enabled the measurement of key physiological traits such as growth rate, nutrient uptake kinetics, temperature and light optima, and allelopathic potential (Labrie et al., 2013; Pittera et al., 2014; Hunter-Cevera et al., 2016; Browning et al., 2017; Fernández-González et al., 2020). These traits, when accurately characterized across representative pigment types and genotypes, can be incorporated into size- or trait-based ecological models (e.g., NEMURO, CoSiNE model) to simulate clade-specific responses to variable environmental drivers (Xiu and Chai, 2014; Gao et al., 2022). In highly dynamic coastal seas like Laizhou Bay, where seasonal forcing interacts with episodic hydrological inputs, such models offer a powerful tool for disentangling the nonlinear interactions shaping picocyanobacterial community assembly and for guiding coastal management under changing climatic conditions.

## 5 Conclusion

Our study reveals that *Synechococcus* communities in Laizhou Bay undergo pronounced seasonal succession, characterized by shifts in both pigment types and genetic clades. Cell abundance peaked in summer, with PE-rich type cells and thermophilic genotypes dominating during *Synechococcus* bloom episodes, especially in the warmer southern region. A critical finding is the identification of an apparent thermal threshold (~ 26°C), beyond which *Synechococcus* abundance increased exponentially and community composition diversified rapidly. This highlights temperature as a primary ecological driver, with riverine nutrient accumulation from summer to autumn likely sustaining *Synechococcus* bloom persistence and contributing to genotypic complexity. Clade turnover, particularly the rise of S5.2. VII and decline of winter-dominant lineages, reflects the community’s dynamic adaptation to seasonal environmental regimes. These findings not only expand our understanding of picocyanobacterial bloom ecology in temperate coastal seas but also underscore the potential for more frequent and intense *Synechococcus* blooms under future warming scenarios. Given the complex and still poorly understood nature of *Synechococcus* population dynamics, this work provides a valuable reference for disentangling and predicting microbial community shifts under the combined pressures of climate change and human disturbance.

## Data Availability

The original contributions presented in the study are included in the article/Supplementary material, further inquiries can be directed to the corresponding authors.
