# Supplementary figures and images for: Seasonal blooms of Synechococcus in a temperate semi-enclosed bay: linking community succession to thermal and nutrient regimes
Source: Front Microbiol. 2025 Aug 5;16:1650890. doi: 10.3389/fmicb.2025.1650890 (PMC12391925; doi:10.3389/fmicb.2025.1650890)

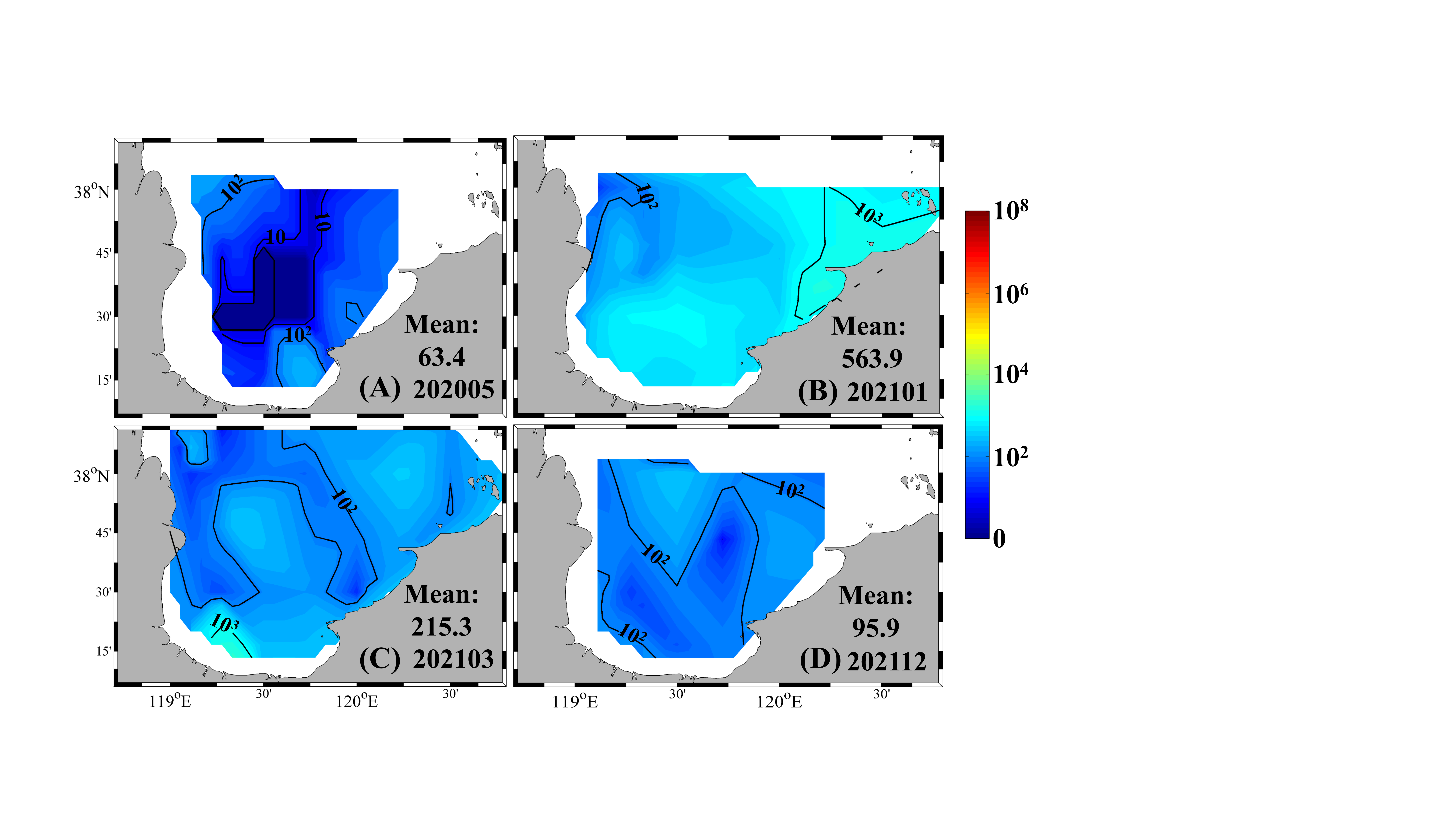

Supplement: Supplementary file 1 [file Image_1.tif]

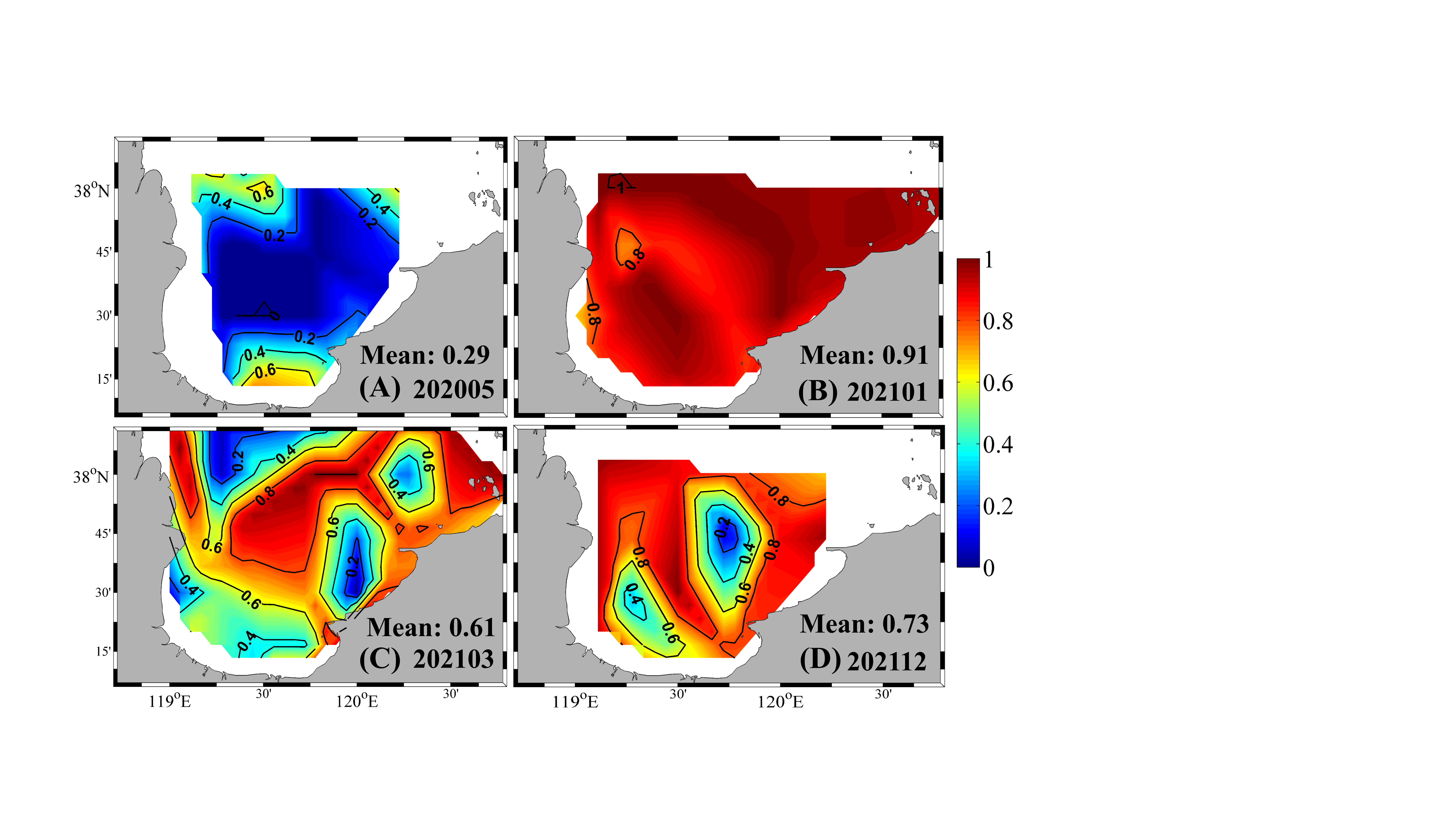

Supplement: Supplementary file 2 [file Image_2.tif]
