# Supplementary material for: Seasonal blooms of Synechococcus in a temperate semi-enclosed bay: linking community succession to thermal and nutrient regimes
Source: Front Microbiol. 2025 Aug 5;16:1650890. doi: 10.3389/fmicb.2025.1650890 (PMC12391925; doi:10.3389/fmicb.2025.1650890)
Supplement: Supplementary file 3 [file Presentation_1.pdf]

Supplementary Material

Captions of Supplementary Figures:

Supplementary Figure 1 *Synechococcus* cell abundance in winter and spring from 2020 to 2022 (unit: cells mL^{-1}).

Supplementary Figure 2 Proportion of PE-type abundance relative to total *Synechococcus* in winter and spring from 2020 to 2022.
