# Supplementary material for: Seasonal blooms of Synechococcus in a temperate semi-enclosed bay: linking community succession to thermal and nutrient regimes
Source: Front Microbiol. 2025 Aug 5;16:1650890. doi: 10.3389/fmicb.2025.1650890 (PMC12391925; doi:10.3389/fmicb.2025.1650890)
Supplement: Supplementary file 4 [file Table_1.docx]

**Supplementary Table S1** Ecological traits of *Synechococcus* clades identified in Laizhou Bay.

| Clade | Ecological traits | References |
| --- | --- | --- |
| S5.1.I | Cold-adapted; associated with nutrient-rich temperate or coastal waters | Farrant et al., 2016; Sohm et al., 2016 |
| S5.1.II | Thermophilic; prefers warm, present in coastal and open-ocean regions | Ahlgren and Rocap, 2012; Kent et al., 2019 |
| S5.1.III | Thermophilic; often co-occurs with S5.1.II in surface blooms | Kent et al., 2019 |
| S5.1.IV | Dominant in cold or temperate coastal waters | Ahlgren and Rocap, 2012; Farrant et al., 2016 |
| S5.1.V | Warm-temperate; estuarine/brackish-capable | Zwirglmaier et al., 2008 |
| S5.1.VI | Warm-temperate; present in coastal and open-ocean regions | Zwirglmaier et al., 2008 |
| S5.1.VII | Warm-temperate; present in coastal and open-ocean regions | Zwirglmaier et al., 2008 |
| S5.1.VIII | Found in hypersaline waters | Ahlgren and Rocap, 2012 |
| S5.1.IX | Nutrient-rich coastal waters | Ahlgren and Rocap, 2012 |
| S5.1.XV | Found in open ocean and East China Sea | Ahlgren and Rocap, 2012 |
| S5.2 | Found in temperate estuaries/coastal waters | Ahlgren and Rocap, 2012 |
| S5.3 | Rarely in marine settings | Ahlgren and Rocap, 2012 |

# References

Ahlgren, N. A. and Rocap, G. (2012), Diversity and distribution of marine *Synechococcus*: Multiple gene phylogenies for consensus classification and development of qPCR assays for sensitive measurement of clades in the ocean, Front Microbiol, 3. doi: 10.3389/fmicb.2012.00213

Farrant, G. K., Doré, H., Cornejo-Castillo, F. M., Partensky, F., Ratin, M., Ostrowski, M., et al. (2016), Delineating ecologically significant taxonomic units from global patterns of marine picocyanobacteria, Proceedings of the National Academy of Sciences, 113(24). doi: 10.1073/pnas.1524865113

Kent, A. G., Baer, S. E., Mouginot, C., Huang, J. S., Larkin, A. A., Lomas, M. W., et al. (2019), Parallel phylogeography of *Prochlorococcus* and *Synechococcus*, The ISME Journal, 13(2), 430-441. doi: 10.1038/s41396-018-0287-6

Sohm, J. A., Ahlgren, N. A., Thomson, Z. J., Williams, C., Moffett, J. W., Saito, M. A., et al. (2016), Co-occurring *Synechococcus* ecotypes occupy four major oceanic regimes defined by temperature, macronutrients and iron, Isme J, 10(2), 333-345. doi: 10.1038/ismej.2015.115

Zwirglmaier, K., Jardillier, L., Ostrowski, M., Mazard, S., Garczarek, L., Vaulot, D., et al. (2008), Global phylogeography of marine *Synechococcus* and *Prochlorococcus* reveals a distinct partitioning of lineages among oceanic biomes, Environ Microbiol, 10(1), 147-161. doi: https://doi.org/10.1111/j.1462-2920.2007.01440.x
